# Garlic-Derived S-allylcysteine Improves Functional Recovery and Neurotrophin Signaling After Brain Ischemia in Female Rats

**DOI:** 10.3390/nu18020362

**Published:** 2026-01-22

**Authors:** Sandra Monserrat Bautista-Perez, Carlos Alfredo Silva-Islas, Maria-del-Carmen Cardenas-Aguayo, Obed-Ricardo Lora-Marín, Maria-del-Carmen Silva-Lucero, Arturo Avendaño-Estrada, Miguel A. Ávila-Rodríguez, Jacqueline V. Lara-Espinosa, Rogelio Hernández-Pando, Martha Menes-Arzate, José Pedraza-Chaverri, Omar Emiliano Aparicio-Trejo, Rosina Sánchez-Thomas, Alejandra Figueroa, Diana Barrera-Oviedo, Perla D. Maldonado

**Affiliations:** 1 Departamento de Farmacología, Facultad de Medicina, Universidad Nacional Autónoma de México, Mexico City 04510, Mexico; sandramo.bape@ciencias.unam.mx (S.M.B.-P.); marzatem@quimica.unam.mx (M.M.-A.); diana.barrera@facmed.unam.mx (D.B.-O.); 2 Departamento de Biología Celular, Facultad de Ciencias, Universidad Nacional Autónoma de México, Mexico City 04510, Mexico; 3 Laboratorio Patología Vascular Cerebral, Instituto Nacional de Neurología y Neurocirugía Manuel Velasco Suárez, Mexico City 14269, Mexico; csilva@innn.edu.mx; 4 Laboratory of Cellular Reprogramming, Departamento de Fisiología, Facultad de Medicina, Universidad Nacional Autónoma de México, Mexico City 04510, Mexico; mcardenasaguayo@unam.mx (M.-d.-C.C.-A.); rk69@ciencias.unam.mx (O.-R.L.-M.); carmenaguila10@hotmail.com (M.-d.-C.S.-L.); 5 Unidad de Radiofarmacia-Ciclotrón, División de Investigación, Facultad de Medicina, Universidad Nacional Autónoma de México, Mexico City 04510, Mexico; arturoae@ciencias.unam.mx (A.A.-E.); avilarod@uwalumni.com (M.A.Á.-R.); 6 Centro de Investigación Sobre el Envejecimiento, Centro de Investigación y de Estudios Avanzados Sede Sur, Mexico City 14330, Mexico; 7 Sección de Patología Experimental, Instituto Nacional de Ciencias Médicas y Nutrición Salvador Zubirán, Mexico City 14080, Mexico; jacqueline.larae@incmnsz.mx (J.V.L.-E.); rogelio.hernandezp@incmnsz.mx (R.H.-P.); 8 Departamento de Biología, Facultad de Química, Universidad Nacional Autónoma de México, Mexico City 04510, Mexico; pedraza@unam.mx; 9 Departamento de Fisiopatología Cardio-Renal, Instituto Nacional de Cardiología Ignacio Chávez, Mexico City 14080, Mexico

**Keywords:** S-allyl cysteine, ischemic stroke, functional nutrition, BDNF, NGF, AKT, ERK

## Abstract

**Background/Objectives:** Ischemic stroke is a leading cause of death and disability, and neuroprotection therapies, or those that increase recovery, are not available. While the garlic-derived bioactive compound S-allyl cysteine (SAC) has shown neuroprotective properties, its subacute long-term effects remain underexplored, particularly in females. **Methods:** We evaluated whether SAC supports functional recovery after ischemia/reperfusion (IR), focusing on neurotrophin signaling, tropomyosin receptor kinase B (TrkB), protein kinase B (AKT), and extracellular signal-regulated kinase (ERK). Adult female Wistar rats underwent 1 h of ischemia and 15 days of reperfusion. SAC (100 mg/kg, i.p.) was administered at the onset of reperfusion and daily for 15 days. Motor and cognitive deficit tests were performed. Infarct area, Ki67, brain-derived neurotrophic factor (BDNF), vascular endothelial growth factor (VEGF), nerve growth factor (NGF), pTrkB, pAKT, and pERK levels were quantified in the cortex, striatum, and hippocampus. **Results:** MicroPET analysis revealed comparable glucose uptake between the IR and IR + SAC groups, indicating similar ischemic severity. SAC reduced infarct area (54.7%) and significantly improved motor deficits (53.9%), circling behavior (38.9%), and long-term memory compared with ischemia/reperfusion (IR) animals. SAC increased the proportion of Ki67-positive cells (4.3-fold in the cortex and 1.8-fold in the striatum) and enhanced neurotrophin levels, NGF (cortex), BDNF (cortex and striatum), VEGF (striatum), pTrkB, pAKT, and pERK (cortex and striatum). **Conclusions:** SAC supports post-ischemic recovery, improving motor performance and preserving long-term recognition memory, effects that could be associated with increased cell proliferation, neurotrophin levels, and activation of the TrkB, AKT, and ERK pathways.

## 1. Introduction

Stroke ranks as the second leading cause of death and the third leading cause of death and disability combined [1]. In 2021, there were 11.9 million new stroke cases and 93.8 million existing cases; additionally, there were 6.55 million deaths. Particularly in 2019, the estimated number of existing stroke cases was higher among females, accounting for 59% [1,2]. Additionally, women experienced poorer outcomes in the activities of daily living, anxiety, and health-related quality of life in both physical and mental aspects [3]. Globally, ischemic stroke accounted for 62.4% of all new stroke cases [4], and it often causes motor and neuropsychiatric problems, including depression and anxiety [5], with about one-third of survivors experiencing memory loss [6,7], which impacts their quality of life and recovery [6,7].

In the ischemic stroke, the vascular occlusion blocks the delivery of glucose and oxygen, resulting in energy failure and ionic imbalance and promoting excitotoxicity, oxidative stress, and inflammation, leading to cell death [8]. Without prompt treatment, ischemic damage worsens [9]. Currently, recombinant tissue plasminogen activator (rTPA) is the only approved pharmacological treatment aimed at restoring cerebral blood flow. However, the percentage of patients who receive this therapy is remarkably low, approximately 1–5% [10,11,12,13]. Furthermore, there are still no effective neuroprotective treatments or therapies that actively promote neural repair [14].

In response to brain injury, the central nervous system activates structural and functional adaptive mechanisms, including neurogenesis, synaptogenesis, and angiogenesis, to support collateral circulation [15,16,17]. These mechanisms are regulated by neurotrophic factors (as neurotrophins) [18], which bind to specific receptors, promoting neurite growth, cell differentiation, and survival [19]. In brain ischemia/reperfusion (IR) models, neurotrophins, such as brain-derived neurotrophic factor (BDNF), vascular endothelial growth factor (VEGF), and nerve growth factor (NGF), activate their receptors, which in turn stimulate AKT and ERK pathways, supporting neuronal survival, differentiation, plasticity, and neurogenesis [20,21,22]. Moreover, neurological deficits are associated with decreased BDNF/tropomyosin receptor kinase B (TrkB) signaling following brain IR injury [20,23,24]. Consequently, stimulating these pathways in the long term represents a therapeutic target.

S-allyl cysteine (SAC), an organosulfur amino acid derived from garlic, exhibits neuroprotective effects following ischemic injury, which are associated with its antioxidant and anti-inflammatory properties [25,26]. However, a poorly understood property of SAC is its neurotrophic effect [25], which is one of the objectives of this work.

Antioxidants can reduce reactive oxygen species (ROS) levels, thereby creating conditions that favor neurogenesis and neuroplasticity [27,28]. In the case of SAC, this compound may lower ROS levels and promote structural and functional plasticity in neural models, including increased axonal branching [29] and enhanced neurite/dendritic complexity in hippocampal neuron cultures [30]. In muscle ischemia, an increase in neovasculogenesis was observed, supporting a proliferative and pro-repair profile of SAC [31]. Also, in aged mice, SAC enhances cell proliferation in the dentate gyrus (as assessed by Ki67, a nonspecific proliferative marker) [32] and, in accelerated-aging or neuroinflammatory/neurodegenerative models, improves memory and upregulates the level of synaptic plasticity—related proteins [30,33,34]. Mechanistically, evidence in neural (cortical slices) and non-neuronal (colonic mucosal epithelial cells, keratinocytes, and HepG2 cells) preparations indicates that SAC can activate pro-survival pathways (ERK and AKT) linked to recovery, preserve BDNF levels, and induce Nrf2/antioxidant response element (ARE) pathway under excitotoxic stress [35,36,37,38]. However, the potential of SAC to modulate neurotrophin signaling during long-term recovery after brain IR remains unexplored.

In addition, the effects of many compounds, including SAC, have not been evaluated in female animals subjected to brain IR, and including female rats is biologically relevant. In young females, sex hormones increase neurotrophin expression and post-ischemic plasticity, all of which may influence recovery after stroke; in contrast, in aged females, the diminution of hormone levels could lead to worse outcomes. Therefore, evaluating these mechanisms in females is necessary to contribute to the development of more inclusive therapeutic strategies [39].

Long-term functional recovery remains a significant clinical need after ischemic stroke, as many survivors experience persistent motor and cognitive deficits. For this reason, the subacute time point selected in this study allows to evaluate recovery-related mechanisms induced by SAC beyond acute neuroprotection, as functional and molecular processes associated with plasticity, repair, and long-term neurological improvement are present.

Nutraceutical and functional nutrition approaches have gained interest as adjuvant strategies to support recovery processes, although mechanistic and preclinical evidence remain limited. SAC is an attractive candidate due to its high bioavailability in preclinical pharmacokinetic studies, overall tolerability in humans when supplemented with aged garlic, and low toxicity in animals after 30 days of consumption [25].

Therefore, the primary aim of the present work was to assess survival, body weight, infarct area, motor deficit, circling behavior, anxiety- and depression-like behaviors, as well as memory after brain IR. As a secondary aim, we evaluate the effect of SAC on proliferation, neurotrophin levels (BDNF, NGF, and VEGF), and survival pathways (pTrkB, pAKT, and pERK). We hypothesized that SAC administration at the onset of reperfusion would support functional recovery by enhancing neurotrophin-mediated signaling and plasticity-related processes during the subacute post-ischemic period.

## 2. Materials and Methods

Based on the aims of this study, outcome measures were predefined and organized hierarchically into primary and secondary endpoints. Functional and structural outcomes were prioritized to assess post-ischemic recovery, while molecular and cellular analyses were included to investigate the underlying mechanisms. A detailed description of all endpoints is provided below.

### 2.1. Animals

Fifty-six female Wistar rats, aged 24–26 weeks, were sourced from the animal care facility of the Facultad de Medicina, Universidad Nacional Autónoma de México, and the Instituto Nacional de Neurología y Neurocirugía. All procedures performed on the animals were carried out in a way that avoided causing them suffering. To this end, the animals were handled by trained personnel. Furthermore, the rats were group-housed (3 per cage) at 22 ± 2 °C, 50–60% humidity, with a 12:12 h light–dark cycle, and provided with food and water ad libitum, along with environmental enrichment. They underwent a 7-day acclimation period before surgery. The estrous cycle was monitored 4 days before surgery. Animals in the metestrus phase of their estrous cycle were selected for surgery.

All procedures were conducted in accordance with institutional and national guidelines, NOM-062-ZOO-1999 [40], the National Institute of Health Guide for the Care and Use of Laboratory Animals, and the guidelines of the Animal Research: Reporting of In Vivo Experiments (ARRIVE). Protocols were approved by the Institutional Animal Care and Use Committee (IACUC) of the Facultad de Medicina, Universidad Nacional Autónoma de México (project 038-CIC-2023), and of the Instituto Nacional de Neurología y Neurocirugía (project 84/20).

### 2.2. Experimental Design

The number of animals used was in accordance with the principles of the 3Rs (Replacement, Reduction, and Refinement) for the use of laboratory animals, ensuring a sufficient number to achieve statistical significance.

Animals were randomly divided into groups, using the simple random allocation method, before surgery as follows:

(1) SHAM, animals subjected to the dissection procedure without occlusion and treated with isotonic saline solution (ISS, vehicle).
(2) SAC, animals subjected to the dissection procedure without occlusion and treated with SAC.
(3) IR, animals subjected to 1 h of ischemia and 15 days of reperfusion, and treated with ISS.
(4) IR + SAC, animals subjected to 1 h of ischemia and 15 days of reperfusion, and treated with SAC.

Animals were monitored for estrous cycle stage, and only females in the metestrus phase were included to minimize hormonal variability, as estrogen levels are lower [41]. Because the reperfusion period spanned 15 days, animals inevitably cycled through additional estrous phases; the estrous phase was standardized at baseline (surgery) and was not controlled throughout the reperfusion period. Following selection, animals were randomly allocated to the experimental groups (SHAM, SAC, IR, and IR + SAC) using a simple randomization procedure based on a random number generator. No block randomization or weight-based stratification was applied.

**Figure 1:**
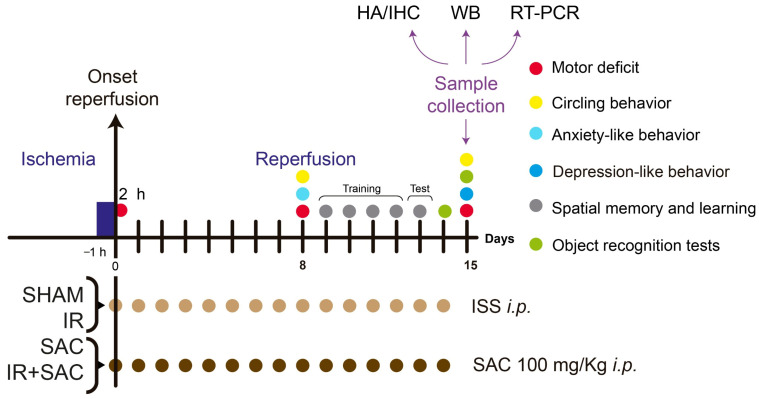
Experimental design. The motor and cognitive tests, sample collection, and the administration schedule for the different groups (SHAM, SAC, IR, and IR + SAC) are shown. IHC: immunohistochemistry; HA: histological analysis; i.p.: intraperitoneal; IR: ischemia/reperfusion; ISS: isotonic saline solution; RT-PCR: reverse transcription polymerase chain reaction; SAC: S-allyl cysteine; WB: Western blot.

Animals in the SAC and IR + SAC groups received SAC (100 mg/kg) intraperitoneally (i.p.) daily for 15 days. This dose was selected based on previous cerebral IR studies in rats, in which this dose of SAC significantly reduced infarct size, oxidative damage, and neurological deficits without evident toxicity [25]. The first dose of SAC was administered at the onset of reperfusion and was given once daily for 15 days. The SAC solution was prepared by dissolving the compound in ISS weekly, kept refrigerated at 4 °C, and protected from light. Meanwhile, the SHAM and IR groups received an equivalent volume of the ISS, administered according to the same schedule as SAC. The overall experimental workflow is summarized in Figure 1 and was designed in accordance with the ARRIVE guidelines to provide a clear chronological framework for the study.

Tissue samples from the 56 animals were collected 15 days after the onset of reperfusion and allocated for histological analysis (HA)/immunohistochemistry (IHC), Western blot (WB), and reverse transcription polymerase chain reaction (RT-PCR) (Table 1). A formal a priori power calculation was not performed. Sample sizes were determined based on prior effect from the literature employing the same ischemia/reperfusion model and comparable behavioral, HA, and molecular endpoints, while adhering to the principles of the 3Rs, particularly reduction.

Differences in sample size among experimental techniques reflect the specific technical requirements, tissue availability, and analytical constraints associated with each assay. For each method, the corresponding *n* value is explicitly defined as the number of animals, sections, analyzed fields, or technical replicates, as indicated below. Before euthanasia, animals underwent a battery of behavioral assessments, including motor deficit, apomorphine-induced circling behavior, anxiety-like behavior, depressive-like behavior, spatial memory, recognition memory, and learning. The specific time points at which each test was performed are indicated in Figure 1. Behavioral tests were conducted following a predefined sequence, with sufficient intervals between tests to reduce fatigue, stress, and potential cross-learning effects. The order of tests was selected to progress from less to more demanding tasks. Before behavioral testing, all animals were habituated to the experimental room and handling procedures to minimize stress-related variability. Behavioral and motor tests were conducted while avoiding auditory and olfactory stimuli.

Additionally, survival, body weight, and infarct area were measured, and the levels of BDNF, VEGF, NGF, TrkB, pTrkB, pAKT, pERK, and Ki-67 were quantified.

All behavioral testing, HA/IHC quantification, molecular analyses (WB and RT-PCR), micro positron emission tomography (microPET), and statistical evaluation were performed by investigators blinded to group allocation using coded sample/animal identifiers. Blinding was maintained throughout data acquisition and analysis and was only broken after the datasets were finalized, to minimize observer and analytical bias.

### 2.3. SAC Synthesis

SAC was synthesized into a round-bottomed flask, equipped with a mechanical stirrer, where L-cysteine hydrochloride monohydrate (30 mmol) and absolute ethanol (90 mL) were added. After 5 min of stirring, sodium (11 mmol) was added in several portions over 30 min to the vigorously stirred suspension. Next, allyl bromide (31 mmol) was incorporated, and the mixture was stirred for one hour. Finally, cold water (30 mL) was introduced to obtain a colorless solution. The ethanol was distilled under reduced pressure, and the solution was cooled in an ice bath. SAC was precipitated with 2.5 mL of acetic acid (final pH 5.6). The white compound was filtered and vacuum-dried. An analytical sample was obtained by crystallization from absolute ethanol for its characterization and semi-quantitative analysis by infrared spectroscopy compared with a SAC standard [42].

### 2.4. Middle Cerebral Artery Occlusion (MCAO)

Anesthesia was induced and maintained with 3% isoflurane using a SomnoSuite vaporizer (Kent Scientific Corporation, Torrington, CT, USA). The ischemia (1 h) was performed by the insertion of a nylon monofilament with a silicone-rubber coating (Doccol Corporation, Sharon, MA, USA) into the common carotid artery until the middle cerebral artery. After one hour of ischemia, the filament was removed, initiating reperfusion (15 days) [43]. Body temperature was maintained during surgery using a warming pad. After surgery, animals received subcutaneous injections of enrofloxacin (10 mg/kg every 24 h, three doses) and meloxicam (3 mg/kg every 24 h, three doses). The first doses were administered at the onset of reperfusion.

### 2.5. Survival and Body Weight Assessment

Following surgery, animals were monitored daily to record survival rates. Survival percentages were plotted over time for each group. Data are presented as the percentage of survival.

Body weight was measured in the same animals at three time points: before surgery, 8 days, and 15 days post-surgery. Measurements were recorded in grams (g).

### 2.6. Micro Positron Emission Tomography (microPET) Imaging and Analysis

The rats were fasted for at least 12 h before the 2-[^18^F]fluoro-2-deoxy-D-glucose ([^18^F]FDG) injection, and glucose levels were measured before radiopharmaceutical administration (113.5 ± 13 mg/dL). Twelve hours after reperfusion, images of each animal were obtained using a MicroPET Focus 120 System (Siemens/CTI, Knoxville, TN, USA) located in the Preclinical MicroPET Research Laboratory at the Faculty of Medicine, National Autonomous University of Mexico. Each animal underwent a microPET scan with [^18^F]FDG. The radiopharmaceutical was administered intravenously via a bolus injection into the tail vein (18.5 MBq ± 4.6) while the animal was under anesthesia induced with 5% isoflurane. Forty minutes after administration, a 10-min brain scan was performed with the animal under anesthesia (2% isoflurane). At the same time, physiological parameters (temperature, heart rate, respiratory rate, oxygen saturation, and blood pressure) were monitored and controlled using a Physiological Monitoring System (Harvard Apparatus). Images were reconstructed using a 2D Ordered Sets Expectation-Maximization algorithm (2D-OSEM) on a 128 × 128-pixel matrix. Then, the images were spatially normalized to the Magnetic Resonance Imaging (MRI) anatomical atlas [44], as implemented in PMOD software (version 4.3, PMOD Technologies LLC, Fällanden, Switzerland). The standardized uptake value (SUV) was computed for all the segmented regions in the atlas. The data from each brain region were analyzed, and the [^18^F]FDG uptake (SUV-ratio: ipsilateral side of the lesion/contralateral side) was determined for all areas. Acquisition post-reperfusion was intended as a quality-control measure of lesion severity. To ensure the correct selection of ROIs, the images were spatially normalized to a magnetic resonance atlas, and to avoid partial-volume effects, only relatively large brain structures were evaluated (Striatum, Cortex, Hippocampus, Thalamus, Cerebellum, Hypothalamus, Amygdala, Olfactory Bulb, and Midbrain).

### 2.7. Assessment of Motor Function

Five different tests were performed to assess motor function at 2 h, 7 days, and 15 days. For each one, we assigned a value of 0 (normal function) or 1 (altered function) (Table 2). The values for each test were added, and a final score was obtained [45].

### 2.8. Apomorphine-Induced Circling Behavior

The cycling behavior was evaluated using apomorphine as previously described [44]. Briefly, apomorphine (1 mg/kg) was administered subcutaneously to each animal. Five minutes after injection, animals were individually placed in separate cages for 1 h. The number of ipsilateral rotations was manually quantified based on video recordings. A rotation was defined as a complete 360° turn along the animal’s longitudinal axis, starting from a clearly defined initial body position.

The test was conducted on the same animals at 8 and 15 days post-surgery. Environmental conditions were controlled throughout the procedure, with standardized lighting and minimized auditory stimuli. Data are expressed as the number of ipsilateral rotations/h.

### 2.9. Histological Analysis

#### 2.9.1. Sample Collection

The animals were anesthetized with intraperitoneal sodium pentobarbital, then perfused transcardially with 100 mL of cold ISS containing heparin (10 U/mL), followed by 100 mL of cold 4% paraformaldehyde (PAF) in phosphate buffer. The brain tissue was refrigerated in PAF solution for 48 h. Subsequently, the samples were dehydrated by placing the tissue in alcohol solutions for 30 min at the following concentrations: 60%, 70%, 80%, 96%, and 100%. The tissue was then placed in alcohol-xylene, then in xylene, and finally in paraffin. Each tissue was embedded in paraffin and sectioned using a microtome [45].

#### 2.9.2. Hematoxylin and Eosin (H&E) Staining

Coronal brain sections (5 μm thick) were deparaffinized in xylene for 30 min, then rehydrated through a descending ethanol gradient. Sections were stained with hematoxylin and rinsed with distilled water. Differentiation was achieved using a saturated lithium carbonate solution, followed by a rinse with distilled water. Slides were then stained with eosin, rinsed again with distilled water, and dehydrated through an ascending ethanol series: 96% ethanol, 100% ethanol, and ethanol–xylene (1:1). Finally, the slides were cleared in xylene and mounted with Entellan resin (Merck, Darmstadt, Hesse, Germany). Histological analysis was performed using a Nikon E200 microscope (Nikon, Melville, NY, USA) [45].

#### 2.9.3. Nissl Staining

Coronal brain sections (5 μm thick) were first deparaffinized in xylene for 30 min, then immersed twice in 100% ethanol. Subsequently, the sections were stained with 0.2% cresyl violet acetate solution. After staining, slides were rinsed with distilled water and sequentially dehydrated in 70% ethanol, 96% ethanol, ethanol–xylene (1:1), and finally xylene. The slides were then mounted using Entellan resin (Merck, Darmstadt, Hesse, Germany). Sections were visualized under a Nikon E200 microscope (Nikon, Melville, NY, USA) [45].

#### 2.9.4. Infarct Area

Sections stained with H&E and Nissl were used to obtain serial images with the microscope’s image-acquisition software, (Image-Pro^®^ Insight 9, Rockville, MA, USA) which captured multiple high-magnification fields covering the ipsilateral hemisphere and automatically stitched them into panoramic images, allowing detailed visualization of cellular morphology throughout the affected region. On these digitized mosaics, the infarct area was manually delineated by drawing to identify cells exhibiting morphological alterations (e.g., pyknotic nuclei or nuclear loss of morphology) and undamaged cells (penumbra). Data are expressed as the percentage of the infarcted area relative to the total area of the ipsilateral hemisphere [45].

### 2.10. Immunohistochemistry (IHC)

The procedures were followed as described by Silva-Islas et al. (2019) [46]. Briefly, slides were deparaffinized in xylene and then rehydrated through a series of descending ethanol solutions and distilled water. After, permeabilization was performed using a 0.15 M phosphate buffer containing 0.2% Triton X-100. Slides were boiled in a 10 mM sodium citrate buffer (pH 6), plus 0.2% Triton X-100. The endogenous peroxidase activity was inactivated with 1% H_2_O_2_. Sections were blocked with 2.5% normal horse serum and incubated overnight at 4 °C with the primary antibodies diluted 1:100: anti-ki67 (Abcam: ab16667; Cambridge, UK), anti-VEGF (Novus Biologicals: NB100664; Centennial, CO, USA), anti-NGF (Bioss Antibodies: bs-067R; Woburn, MA, USA), and anti-BDNF (Novus Biologicals: NB10098682). Then, the Vectastain Universal Quick HRP kit (PK-6200, Newark, CA, USA) was used according to the manufacturer’s instructions, followed by incubation with 3,3′-diaminobenzidine and counterstaining with hematoxylin. For histological analysis, images of the striatum (12 images) and cortex (8 images) were acquired from each coronal brain section per animal. Immunopositive cells were identified based on staining intensity, clearly distinguishable from the background signal. We also performed a negative control using a sample without primary or secondary antibodies. To minimize inter-slide variability, all sections from the same brain region were processed under identical staining conditions, and signal intensity was normalized within each slide before quantification. In each one, the total number of cells and the number of positive cells for each marker (cells that exhibited brown staining) were counted. Data are expressed as the percentage of positive cells for each marker.

### 2.11. Western Blot (WB)

The cortex, striatum, and hippocampus of each rat were homogenized to make 10% (*w*/*v*) homogenate. The pre-chilled homogenization buffer contained 50 mM Tris—HCl (pH 7.4), 8.5% sucrose, 2 mM EDTA, 2 mM EGTA, 10 mM β-mercaptoethanol plus the following protease and phosphatase inhibitors: 0.5 mM AEBSF, 10 μg/mL aprotinin, 10 μg/mL leupeptin, 4 μg/mL pepstatin, 5 mM benzamidine, 20 mM beta-glycerophosphate, 50 mM sodium fluoride, 1 mM sodium orthovanadate, and 100 nM okadaic acid. Protein concentration was determined by the modified bicinchoninic acid Pierce BSA protein assay kit (23225, Thermo Scientific, Waltham, MA, USA). The tissue homogenates were boiled in Laemmli’s buffer for 5 min and then subjected to 10% and 15% SDS-polyacrylamide gel electrophoresis (PAGE). Proteins were transferred to the 0.45 μm Immobilon-P membrane (Millipore, Bedford, MA, USA). After transfer, the membranes were blocked with serum albumin for 1 h and incubated with the following primary antibodies related to the neurotrophin signaling pathway: anti-pTrkB 1:1000 (Santa Cruz Biotechnology: sc135645; Dallas, TX, USA), anti-TrkB 1:1000 (Santa Cruz Biotechnology: sc8316), anti-pAKT 1:1000 (Genetex: gtx 50120; Irvine, CA, USA), anti-pERK 1:500 (Cell Signaling Technology: 4370P; Danvers, MA, USA), anti-VEGF (Genetex: GTX102643), BDNF 1:1000 (Merck: GF35L-100; Darmstadt, Germany), anti-HPRT 1:1000 (hypoxanthine-guanine phosphoribosyl transferase) (Genetex: GTX63074), and anti-GAPDH 1:1000 (glyceraldehyde-3-phosphate dehydrogenase) (Genetex: GTX627408) for 24 h. The membranes were washed with TBS-T buffer (20 mM Tris Base, 150 mM NaCl, and 0.1% Tween 20). Subsequently, the membranes were incubated with the corresponding secondary antibody diluted 1:10,000 (Jackson ImmunoResearch Laboratories, goat anti-mouse: AB_2338457; or goat anti-rabbit: AB_2337910, West Grove, PA, USA) for 1 h and washed with TBS-T to remove excess secondary antibody. Finally, the membranes were visualized using a chemiluminescence reaction (Immobilon Chemiluminescent HRP High Sensitivity Substrate, Sigma-Aldrich, St. Louis, MO, USA). The protein bands were scanned and imaged using the ChemiDoc XRS+ system (Bio-Rad, Hercules, CA, USA) and analyzed with Image Lab 6.1 software (Bio-Rad). HPRT and GAPDH were used as reference proteins depending on the brain region and target protein analyzed. The selection was based on their reported stability under brain IR conditions and on preliminary assessments confirming consistent expression across experimental groups in each tissue using total protein stain (Ponceau). To quantify protein levels, each immunoreactive band was normalized to its corresponding band. Data are presented as the optical density ratio of the BDNF/HPRT, VEGF/HPRT, pTrkB/TrkB, pAKT/GAPDH, pERK/GAPDH, pAKT/HPRT, and pERK/HPRT (*n* = 2 technical duplicates) [47].

### 2.12. Reverse Transcription Polymerase Chain Reaction (RT-PCR)

Cortex, striatum, and hippocampus were dissected from the animals, and the tissue was placed in a buffer containing guanidine thiocyanate (RNeasy Mini kit, Qiagen, Hilden, Germany) and β-mercaptoethanol to lyse the cells and prevent RNA denaturation. Samples were placed under nitrogen and stored at −70 °C. Subsequently, the tissue was homogenized with silica and zirconia beads and centrifuged. The supernatant was recovered, and 70% ethanol was added. The mixture was then placed on a silica column and centrifuged. The filtrate from this column was discarded, and the column contents were washed with the manufacturer’s buffers. Subsequent centrifugations were performed using the RNeasy Mini kit (Qiagen). Finally, RNase-free water was added to separate the RNA from the column. The quantity and purity of the RNA sample were assessed by spectrophotometry (A260/A280) using a Nanodrop (Thermo Scientific, Waltham, MA, USA). One hundred nanograms of RNA were used to produce cDNA via reverse transcription according to the instructions of the Omniscript kit (Qiagen, Hilden, Germany). The primer sequences for qRT-PCR are listed in Table 3. Initial denaturation at 95 °C for 15 min was followed by 40 cycles of amplification, each consisting of 95 °C for 20 s, 60 °C for 20 s, and 72 °C for 34 s (7500 Real-time PCR System, Applied Biosystems, Foster City, CA, USA). Each sample was examined twice. The 2^−∆∆Ct^ technique calculates the fold change in gene expression. Relative mRNA is presented as relative expression values normalized to beta-actin (ACTB) and GAPDH, selected as reference genes for RT-PCR normalization based on their widespread use and reported stability in brain tissue [48].

### 2.13. Anxiety-like Behavior I Elevated Maze

Anxiety-like behavior was assessed via the I-maze [49], which consists of a straight corridor (resembling the letter I) divided into two “closed” arms and one “open” segment. The animals were placed in an I-shaped device for 5 min for this test. The percentage of time spent in the open segment and the number of head dips into the open zone are inversely associated with anxiety-like behavior. Conversely, more entries into closed arms and a higher frequency of stretch–attend postures reflect elevated anxiety-like behavior. Behavioral outcomes are reported as the percentage of time spent in the open field, number of head dips into the open and closed arms, and number of stretch-attend postures, respectively. The test was performed 7 days after surgery to assess the effects of the recovery process and behavioral alterations [50].

### 2.14. Depression Like Behavior

To evaluate depression-like behavior, animals were suspended by the tail for 5 min. During this test, the time spent immobile was quantified as an indicator of depression-like behavior. Immobility is reported as the percentage of time the animal remained motionless [51]. This test was performed 15 days after surgery to avoid interfering with the memory studies [52].

### 2.15. Object Recognition Tests

An open black PVC box (70 × 50 × 60 cm) and two identical objects (cleaned with 70% alcohol) were used. The objects were placed in opposite corners, 10 cm from the walls. The animals received a 5-min habituation session in the box without objects. One hour later, each animal was placed with two identical objects for 5 min, and 1 h later, with one different object placed at random for another 5 min. Twenty-four hours later, the animals were placed with a third, different object for 5 min. The time spent with the new and known objects was quantified. Recognition memory performance is expressed as the ratio of novel object interactions/total interactions, reported as the recognition index [53].

### 2.16. Spatial Memory and Learning

Four training sessions were carried out daily. In each training, the rat was introduced into the maze from a different starting point around the pool. The animals were left swimming and exploring for 1 min; after this time, if they did not find the platform, they were directed to it and left there for 15 s. The time (in s) each animal took to reach the platform was quantified. On the fifth day, the platform was removed, and the animals were reintroduced. We quantify how long they remained in the area around the platform during training. Data are expressed as the percentage of time spent in the target quadrant where the platform had previously been located.

### 2.17. End Points

Primary endpoints included functional and structural outcomes, such as infarct area (15 days), survival (0–15 days), body weight (0, 8, and 15 days), motor deficit (2 h, 8, and 15 days), circling behavior (8 and 15 days), anxiety-like (8 days) and depression-like (15 days) behaviors, and memory performance (14 and 15 days) following brain IR.

Secondary endpoints focused on recovery-associated mechanisms, including cell proliferation (Ki67 levels in cortex and striatum), neurotrophins (BDNF and VEGF levels in cortex, striatum, and hippocampus; NGF levels in cortex and striatum), and activation of signaling pathways (pTrkB, pAKT, and pERK levels in cortex, striatum, and hippocampus), evaluated 15 days after brain IR.

### 2.18. Statistical Analysis

Survival data were analyzed using the Log-rank (Mantel-Cox) test. Body weight, motor dysfunction, and circling behavior were analyzed using a two-way mixed-effects model with repeated measures. Time after ischemia (2 h, 8 days, and 15 days) was entered as the within-subject (row) factor and treatment as the between-subject (column) factor (SHAM, SAC, IR, and IR + SAC), with subject included as a random effect. Sphericity was not assumed, and degrees of freedom were corrected using the Geisser–Greenhouse epsilon. When a significant main effect or interaction was detected by Šidák’s or Tukey multiple-comparisons test, it was applied to compare IR vs. IR + SAC at each time point and to assess changes over time within each group (α = 0.05). For the following experiments, differences between groups were evaluated using a one-way ANOVA and Bartlett’s test for homogeneity of variances. When the ANOVA was significant, group differences among the four experimental groups were explored with Tukey’s post hoc multiple-comparison tests (α = 0.05). For key outcomes, we report the *p*-value and the effect size estimate. Data are expressed as mean ± SD. Statistical analysis was performed using GraphPad Prism 10.6.1 software (GraphPad Software, La Jolla, CA, USA). In all cases, a *p* ≤ 0.05 was considered statistically significant.

## 3. Results

### 3.1. SAC Does Not Affect Survival and Body Weight

**Figure 2:**
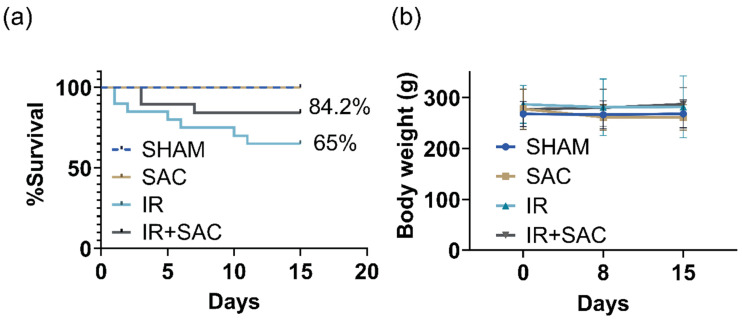
Effect of S-allylcysteine (SAC) on survival and body weight after ischemia/reperfusion (IR) in female rats. Animals underwent 1 h of ischemia followed by 15 days of reperfusion. (**a**) Survival (%) over 15 days of reperfusion. (**b**) Body weight before surgery and at 8 and 15 days of reperfusion. Groups are SHAM and SAC (*n* = 13 animals per group), IR (*n* = 14 animals per group), and IR + SAC (*n* = 16 animals per group). Data are represented as the mean ± SD (for body weight).

The survival rate in the IR group was 65%, while the IR + SAC group had a survival rate of 84.2% (Figure 2a). Over the course of the study, 3 animals died in the IR + SAC group (15.85%), compared with 7 animals in the IR group (35%). Kaplan–Meier analysis showed no significant difference in survival between IR and IR + SAC. Moreover, no differences in body weight were observed between groups or across time points (before surgery and at 8 and 15 days) (Figure 2b).

### 3.2. SAC Reduces Infarct Area, Motor Deficit, and Circling Behavior

**Figure 3:**
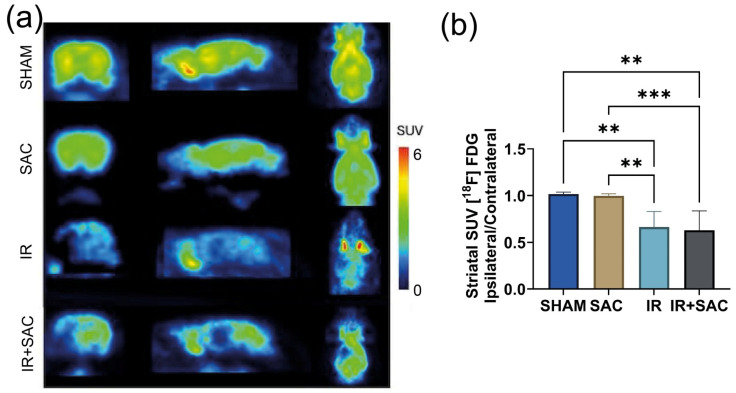
Effect of S-allylcysteine (SAC) on [^18^F]FDG uptake evaluated by microPET after ischemia/reperfusion (IR) in female rats. Animals underwent 1 h of ischemia followed by 15 days of reperfusion. (**a**) Micro PET images (coronal, sagittal, and axial plane axes) were acquired 12 h after reperfusion using [^18^F]FDG. (**b**) Plot of standardized uptake value (SUV) ratio in the striatum [ipsilateral/contralateral]. Groups are SHAM (*n* = 4 animals per group) and SAC, IR, and IR + SAC (*n* = 7 animals per group). Data are represented as the mean ± SD. ** *p* < 0.01 and *** *p* < 0.001.

[^18^F]FDG microPET imaging allows the evaluation of glucose consumption in brain cells. In severe ischemic processes with cell death, [^18^F]FDG uptake is considerably reduced; thus, the technique allows quantification of damaged regions through glucose uptake in a non-invasive way. MicroPET images obtained for the different evaluated animal groups are shown in Figure 3a. The lesioned hemispheres of the IR and IR + SAC groups showed a reduction of [^18^F]FDG uptake (when compared with the SHAM and SAC groups) across multiple brain regions, including: nucleus accumbens, amygdala, auditory cortex, entorhinal cortex, cingulate cortex, frontal association cortex, insular cortex, orbitofrontal cortex, medial prefrontal cortex, motor cortex, parietal association cortex, retrosplenial cortex, visual cortex, somatosensory cortex, striatum, anterodorsal hippocampus, hypothalamus, olfactory regions, cerebellar gray and white matter, and thalamus. But the most affected brain region was the striatum (Figure 3b). Furthermore, no significant differences were observed in any evaluated area between the IR and IR + SAC groups, indicating correct occlusion of the middle cerebral artery in all animals in both groups. In the striatal region, the average [^18^F]FDG uptake (SUV ratio) in the damaged area for each group was SHAM (1.02), SAC (0.996), IR (0.633), and IR + SAC (0.629) (Figure 3b).

**Figure 4:**
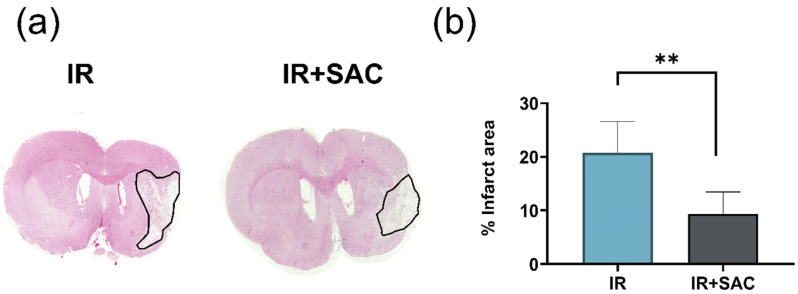
Effect of S-allylcysteine (SAC) on infarct area after ischemia/reperfusion (IR) in female rats. Animals underwent 1 h of ischemia followed by 15 days of reperfusion. (**a**) Representative coronal sections stained with hematoxylin & eosin. (**b**) Quantification of the percentage of infarct area in the affected hemisphere. Groups are: SHAM and SAC (*n* = 3 animals per group), and IR and IR + SAC (*n* = 4 animals per group). Data are represented as the mean ± SD. ** *p* < 0.01.

To assess the extent of tissue damage, the infarct area was quantified using H&E and Nissl staining. SHAM and SAC groups did not show an infarct area, whereas the mean infarct area in the IR group was 20.77% with H&E (Figure 4a) and 19.1% with Nissl (Figure S1a) staining. In contrast, SAC treatment decreased the infarct area in the IR + SAC group to 9.4% (H&E) and 7.2% (Nissl), indicating a smaller infarct than the IR group (Figure 4b and Figures S1b).

**Figure 5:**
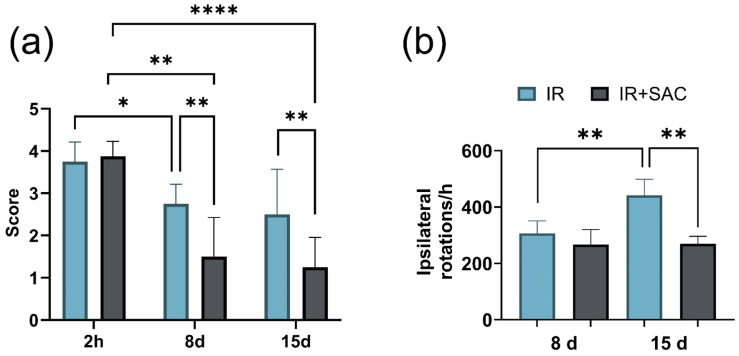
Effect of S-allylcysteine (SAC) on motor dysfunction and circling behavior after ischemia/reperfusion (IR) in female rats. Animals underwent 1 h of ischemia followed by 15 days of reperfusion. (**a**) Motor deficit score (0–5; higher scores indicate greater deficit) at 2 h, day 8, and day 15; SHAM and SAC (*n* = 11 animals per group), IR and IR + SAC (*n* = 8 animals per group). (**b**) Apomorphine-induced circling behavior (1 mg/kg, i.p.); SHAM, SAC, and IR (*n* = 4), IR + SAC (*n* = 5 animals per group). Data are represented as mean ± SD. * *p* < 0.05, ** *p* < 0.01, and **** *p* < 0.0001.

In the motor function, the SHAM and SAC groups scored zero at all time points evaluated post-surgery. Mixed-effects analysis of motor behavior revealed a significant main effect of time: a temporal improvement in IR and IR + SAC groups. In the IR group, scores only decreased modestly between 2 h and 8 days. In contrast, in the IR + SAC group, motor deficit markedly decreased between 2 h and 8 days and between 2 h and 15 days. In addition, motor deficits were similar between groups at 2 h after ischemia, and the IR + SAC group displayed lower motor deficit scores than the IR group at 8 days (IR: 2.75 vs. IR + SAC: 1.50) and 15 days (IR: 2.71 vs. IR + SAC: 1.25) (Figure 5a).

In apomorphine-induced rotational behavior, at 8 and 15 days, the IR group exhibited a higher number of contralateral rotations compared with the SHAM and SAC groups (Figure 5b). Mixed-effects analysis revealed a strong main effect of treatment group on the number of apomorphine-induced rotations at 15 days. On day 8, both ischemic groups displayed marked circling (IR: 306.7 rotations; IR + SAC: 266.8 rotations), but no significant differences were detected between the groups. On day 15, the number of ipsilateral rotations further increased in the IR group (441.6 rotations), and the SAC-treated group (IR + SAC) had a lower mean of 269.8 rotations (Figure 5b).

To evaluate the potential differential neuroprotective effect of SAC in multiple brain regions, we focus our assessments on the cortex, striatum, and hippocampus.

### 3.3. SAC Does Not Modify BDNF and VEGF Levels, but Increases the Content of NGF, p-TrkB, p-AKT, and p-ERK in the Cortex

BDNF, VEGF, and NGF are neuroprotective growth factors. Therefore, we aim to investigate whether SAC can modify the expression of these neurotrophins as part of its protective effects in regions near the site of damage (cortex and striatum) and in an area related to cognitive impairments (hippocampus). Each region is described below.

**Figure 6:**
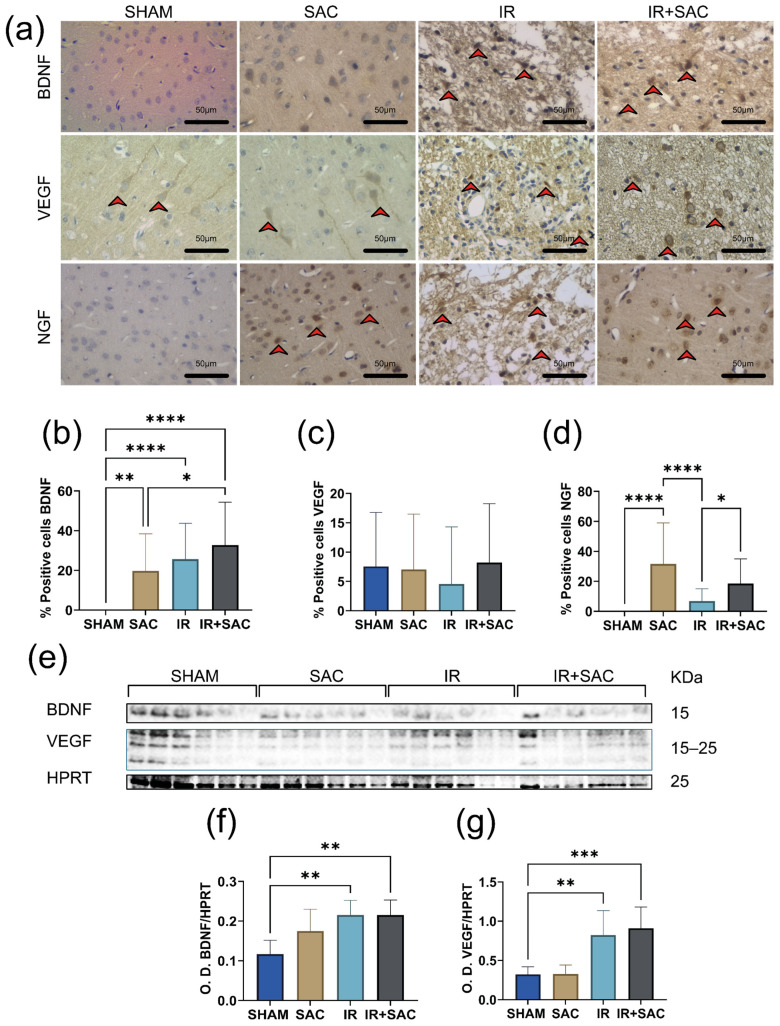
Effect of S-allylcysteine (SAC) on neurotrophin expression in cortex after ischemia/reperfusion (IR) in female rats. Animals underwent 1 h of ischemia followed by 15 days of reperfusion. (**a**) Representative immunohistochemistry (IHC) for brain-derived neurotrophic factor (BDNF), vascular endothelial growth factor (VEGF), and nerve growth factor (NGF). Scale bar: 50 μm. Positive cells are indicated with a red arrow. IHC graph: % positive cells for (**b**) BDNF, (**c**) VEGF, and (**d**) NGF per field. SHAM and SAC (*n* = 3 animals per group), IR (*n* = 4 animals per group), and IR + SAC (*n* = 6 animals per group). (**e**) Representative Western blots for BDNF and VEGF. Hypoxanthine-guanine phosphoribosyl transferase (HPRT) served as a loading control. Densitometric quantification (Optical density: O.D.) for (**f**) BDNF and (**g**) VEGF immunoblots normalized to HPRT. SHAM, SAC, IR, and IR + SAC (*n* = 6 animals per group). Data are represented as mean ± SD. * *p* < 0.05, ** *p* < 0.01, *** *p* < 0.001 and **** *p* < 0.0001.

In the cortex of SHAM animals, no BDNF-positive cells were detected by IHC (Figure 6a,b), although WB quantification confirmed detectable protein levels (Figure 6e,f). SAC treatment alone (SAC group) increased the percentage of BDNF-positive cells (19.72%) compared with the SHAM, as determined by IHC (Figure 6b). Both the IR (25.70%) and IR + SAC (32.79%) groups exhibited elevated BDNF protein levels (by IHC) compared with the SHAM group, but no significant difference observed between the IR and IR + SAC groups (Figure 6b). Notably, cortical BDNF changes were consistent using WB, supporting the robustness of this finding. In contrast, cortical BDNF mRNA expression measured by RT-PCR did not differ among groups (Figure S2a).

By IHC, VEGF protein levels were similar across the groups (Figure 6a,c). In contrast, an increase in cortical VEGF levels was observed in the IR and IR + SAC groups by WB (Figure 6e,g). VEGF mRNA expression increased only in the SAC group (Figure S2b).

SAC treatment markedly increased the proportion of NGF-positive cells in the cortex (SAC group: 31.7% vs. SHAM group: 0%). The IR + SAC group also showed a higher number of NGF-positive cells (18.6%) than the IR group (6.9%; Figure 6a,d) by IHC. NGF mRNA expression increased by 2.7-fold in the IR group and 3.4-fold in the IR + SAC group, compared to the SHAM group (Figure S2c).

**Figure 7:**
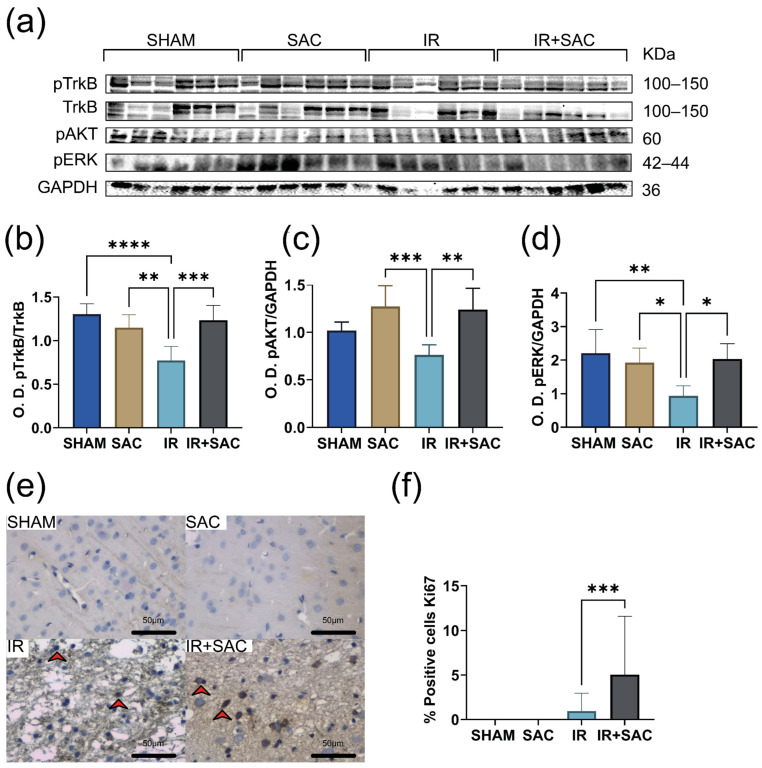
Effect of S-allylcysteine (SAC) on pTrkB, pAKT, and pERK levels and cell proliferation in cortex after ischemia/reperfusion (IR) in female rats. Animals underwent 1 h of ischemia followed by 15 days of reperfusion. (**a**) Representative Western blots for phospho-tropomyosin receptor kinase (pTrkB), tropomyosin receptor kinase B (TrkB), phospho-protein kinase B (pAKT), and phospho-extracellular signal-regulated kinases 1/2 (pERK). TrkB served as a loading control for pTrkB, and glyceraldehyde-3-phosphate dehydrogenase (GAPDH) served as a loading control for pAKT and pERK. Densitometric quantification (Optical density: O.D.) for (**b**) pTrkB/TrkB, (**c**) pAKT/GAPDH, and (**d**) pERK/GAPDH. SHAM, SAC, IR, and IR + SAC (*n* = 6 animals per group). (**e**) Representative immunohistochemistry (IHC) for Ki-67. Scale bar: 50 μm. Positive cells are indicated with a red arrow. (**f**) IHC graph: % positive Ki67 cells. SHAM and SAC (*n* = 3 animals per group), IR (*n* = 4 animals per group), and IR + SAC (*n* = 6 animals per group). Data are represented as mean ± SD. * *p* < 0.05, ** *p* < 0.01, *** *p* < 0.001, and **** *p* < 0.0001.

To identify the signaling pathways underlying the neuroprotective effect of SAC in female rats subjected to IR, we evaluated the phosphorylation levels of TrkB, AKT, and ERK in the cortex. Ischemia markedly reduced the pTrkB, pAKT, and pERK levels. SAC treatment prevented these reductions, restoring phosphorylation levels to levels comparable to those of the SHAM group (Figure 7a–d).

No Ki67-positive cells were observed in the SHAM and SAC groups (Figure 7e,f). In contrast, Ki67-positive cells were observed in the IR (0.95%) and IR + SAC (5.04%) groups.

### 3.4. SAC Increases BDNF Expression and Proliferation, Activating pTrkB, pAKT, and pERK in the Striatum

**Figure 8:**
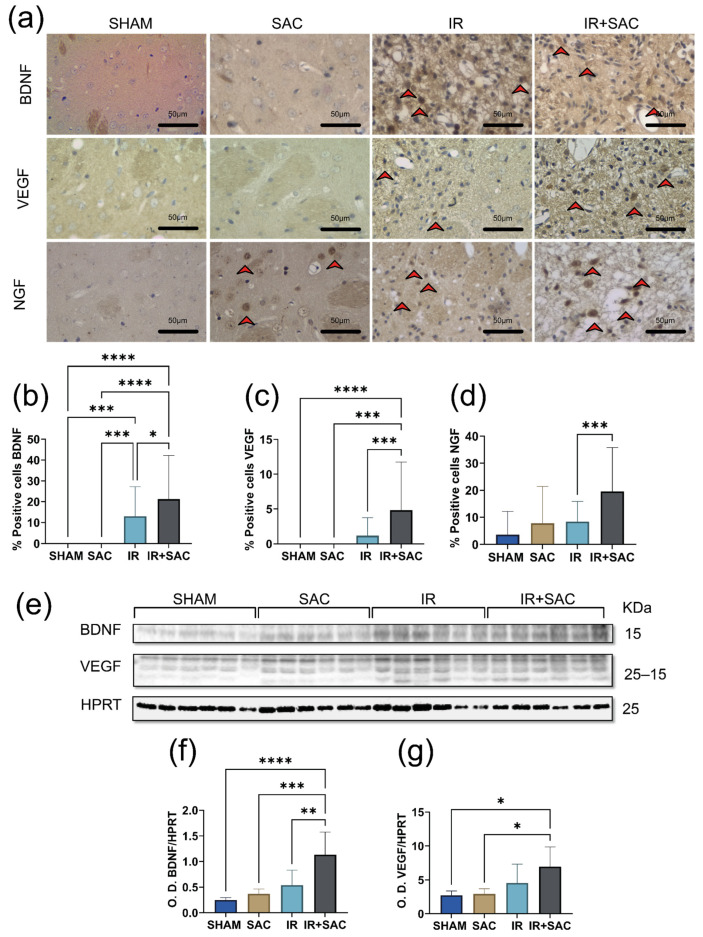
Effect of S-allylcysteine (SAC) on neurotrophin expression in striatum after ischemia/reperfusion (IR) in female rats. Animals underwent 1 h of ischemia followed by 15 days of reperfusion. (**a**) Representative immunohistochemistry (IHC) for brain-derived neurotrophic factor (BDNF), vascular endothelial growth factor (VEGF), and nerve growth factor (NGF). Scale bar: 50 μm. Positive cells are indicated with a red arrow. IHC graph: % positive cells for (**b**) BDNF, (**c**) VEGF, and (**d**) NGF per field. SHAM and SAC (*n* = 3 animals per group), IR (*n* = 4 animals per group), and IR + SAC (*n* = 6 animals per group). (**e**) Representative Western blots for BDNF and VEGF. Hypoxanthine-guanine phosphoribosyl transferase (HPRT) served as a loading control. Densitometric quantification (Optical density: O.D.) for (**f**) BDNF and (**g**) VEGF immunoblots normalized to HPRT. SHAM, SAC, IR, and IR + SAC (*n* = 6 animals per group). Data are represented as mean ± SD. * *p* < 0.05, ** *p* < 0.01, *** *p* <0.001, and **** *p* < 0.0001.

In the striatum, ischemia increased the proportion of BDNF-positive cells. No positive cells were observed in the SHAM and SAC groups. The IR + SAC (21%) group displayed higher BDNF immunoreactivity than IR animals (13%) (Figure 8a,b). By WB, low BDNF levels were observed in the SHAM and SAC groups, and similar changes in BDNF levels were observed in the IR and IR + SAC groups, supporting the robustness of this finding (Figure 8e,f).

No positive cells to VEGF were observed in the SHAM and SAC groups. VEGF-positive cells were more abundant in the IR + SAC group (4.8%) than in the IR group (1.1%) (Figure 8a,c). By WB, low VEGF levels were observed in the SHAM and SAC groups, and similar changes in VEGF levels were observed in the IR and IR + SAC groups, supporting the robustness of this finding (Figure 8e,g).

For NGF, the IR + SAC group (19.6%) showed a higher percentage of immunopositive cells than the IR (8.4%), SHAM (3.56%), and SAC (7.8%) groups (Figure 8d).

In contrast, mRNA analysis showed no significant changes in BDNF, VEGF, or NGF expression in the striatum among groups (Figure S2d–f).

**Figure 9:**
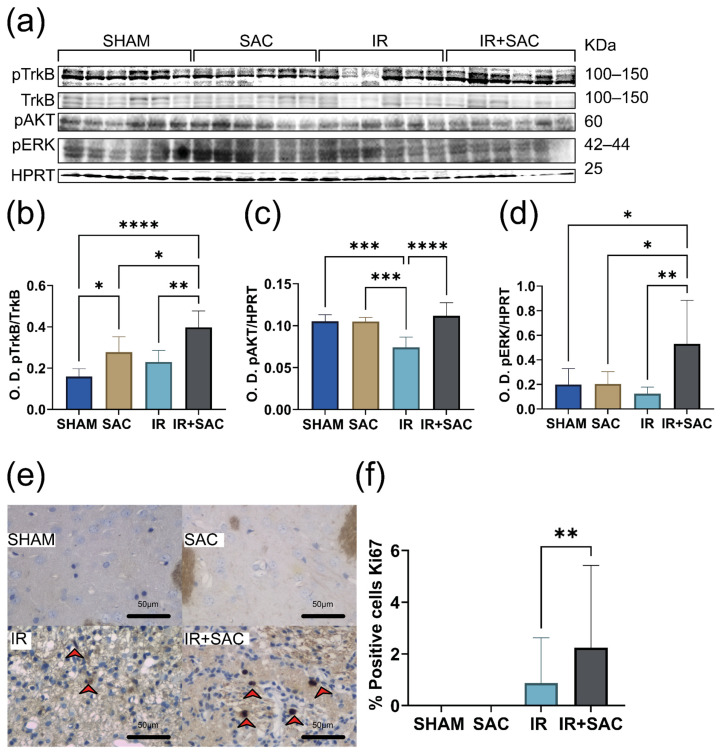
Effect of S-allylcysteine (SAC) on pTrkB, pAKT, and pERK levels and cell proliferation in striatum after ischemia/reperfusion (IR) in female rats. Animals underwent 1 h of ischemia followed by 15 days of reperfusion. (**a**) Representative Western blots for phospho-tropomyosin receptor kinase (pTrkB), tropomyosin receptor kinase B (TrkB), phospho-protein kinase B (pAKT), and phospho-extracellular signal-regulated kinases 1/2 (pERK). TrkB served as a loading control for pTrkB, and hypoxanthine-guanine phosphoribosyltransferase (HPRT) served as a loading control for pAKT and pERK. Densitometric quantification (Optical density: O.D.) for (**b**) pTrkB/TrkB, (**c**) pAKT/HPRT, and (**d**) pERK/HPRT. Densitometric quantification (Optical density: O.D.) for (**b**) pTrkB/TrkB, (**c**) pAKT/HPRT, and (**d**) pERK/HPRT. SHAM, SAC, IR, and IR + SAC (*n* = 6 animals per group). (**e**) Representative immunohistochemistry (IHC) for Ki-67. Scale bar: 50 μm. Positive cells are indicated with a red arrow. (**f**) IHC graph: % positive Ki67 cells. SHAM and SAC (*n* = 3 animals per group), IR (*n* = 4 animals per group), and IR + SAC (*n* = 6 animals per group). Data are represented as mean ± SD. * *p* < 0.05, ** *p* < 0.01, *** *p* < 0.001, and **** *p* < 0.0001.

Analysis of signaling pathways revealed that SAC treatment enhanced TrkB phosphorylation in the striatum. The IR + SAC group exhibited the highest pTrkB/TrkB ratios among all groups (Figure 9a,b). The ischemia reduced pAKT and pERK levels, and SAC treatment blocked this decrease (IR + SAC animals) (Figure 9c,d).

No Ki67-positive cells were detected in the SHAM or SAC groups. SAC (~2.3%) treatment significantly increased the proportion of Ki67-positive cells compared with the IR (~0.8%) group (Figure 9e,f).

### 3.5. SAC Does Not Alter Neurotrophin Expression nor the Levels of pTrkB, pAKT, and pERK in the Hippocampus

In the hippocampus, WB analysis revealed that only ischemia decreased BDNF protein levels (IR group) compared with the SHAM group (Figure S3a,b), without changes in BDNF mRNA expression (Figure S3c). No changes were observed in VEGF protein levels in the groups studied (Figure S3a,d). In fact, VEGF mRNA expression was reduced in SAC-treated and ischemic animals relative to SHAM (Figure S3e).

Phosphorylation of TrkB was increased in ischemic animals (IR and IR + SAC groups) (Figure S3f,g). In contrast, AKT phosphorylation was reduced in SAC, IR, and IR + SAC groups compared with SHAM, with no differences between ischemic groups (Figure S3f,h). ERK phosphorylation increased only in ischemic animals (Figure S3f,i).

### 3.6. IR Does Not Induce Anxiety-like and Depression-like Behavior

IR animals did not show anxiety-like behavior evaluated in the I-maze, as indicated by time spent on the open arm, unprotected head dipping, protected head dipping, and the stretched-attend posture (Figure S4a–d).

No depressive-like behavior was observed using the tail suspension test, quantifying the percentage of immobility time in the SHAM (59.4%), SAC (35.5%), IR (71.78%), and IR + SAC (63.4%) groups.

### 3.7. SAC Enhances Long-Term Recognition Memory, but Does Not Affect Spatial Recognition Memory

**Figure 10:**
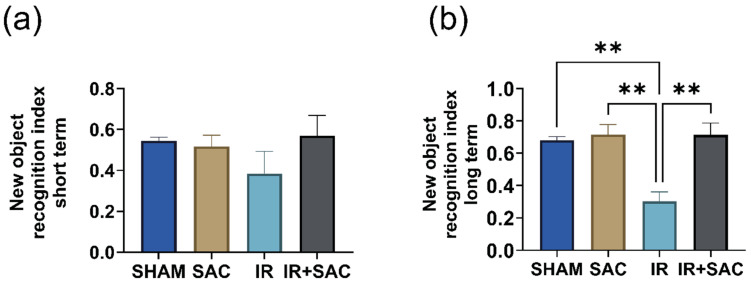
Effect of S-allylcysteine (SAC) on memory after ischemia/reperfusion (IR) in female rats. Animals underwent 1 h of ischemia followed by 15 days of reperfusion. (**a**) Short-term recognition index and (**b**) long-term recognition index. The short-term and long-term object recognition test were performed on day 14 and 15 of reperfusion, respectively. SHAM, SAC, IR, and IR + SAC (*n* = 4 animals per group). Data are represented as mean ± SD. ** *p* < 0.01.

Short- and long-term recognition memory were evaluated using the novel object recognition test. Short-term memory remains unaffected, as recognition indices were 0.52 in SHAM, 0.55 in SAC, 0.38 in IR, and 0.57 in IR + SAC (Figure 10a). In long-term recognition memory, the IR group showed a lower recognition index (0.30) than SHAM and SAC (0.68), and SAC treatment preserved long-term recognition memory after IR in female rats (IR + SAC: 0.72) (Figure 10b).

In the spatial memory and learning task, daily escape latency was recorded (time from the start to reaching the platform). All animals showed a decrease in latency over several days. On day 13, escape latencies and times in the target quadrant did not differ.

## 4. Discussion

In this study, we evaluated the effects of S-allyl cysteine (SAC) in young female rats subjected to transient middle cerebral artery occlusion. SAC post-ischemic treatment reduced infarct size, improved motor deficits (including apomorphine-induced circling behavior), increased the percentage of Ki67-positive cells in the cortex and striatum, and enhanced the phosphorylation of TrkB, AKT, and ERK in these regions. At the level of neurotrophins, SAC increased BDNF, VEGF, and NGF levels in the cortex and striatum and improved long-term recognition memory.

Ischemic stroke is a leading cause of death and disability worldwide and is frequently followed by long-term cognitive and behavioral deficits, including anxiety, depression, and memory impairments [5]. Pharmacological strategies that consistently provide neuroprotection or stimulate recovery are still lacking. A sex-related dimorphism has been described in the epidemiology and long-term outcomes of ischemic stroke [54]. Female brains exhibit higher numbers of microglial cells and increased levels of proinflammatory cytokines, resulting in a predominantly proinflammatory phenotype. Microglial cells are distributed in the parietal cortex, CA1, CA3, dentate gyrus, and amygdala—regions affected by stroke and involved in cognitive functions such as memory [55]. Differences in cell death pathways have also been reported, with caspase-dependent mechanisms predominating in females and PARP-1–mediated cell death in males [55], as well as sex-related differences in the response to pharmacological treatments such as aspirin [56]. Although stroke is more frequent after menopause [57], an increase in younger women has been observed, due to lifestyle changes [58]. Moreover, there are no neuroprotective or regenerative therapies to treat stroke.

In preclinical models of brain IR, the neuroprotective effect of SAC in males has mainly been attributed to its antioxidant properties; however, SAC also exhibits neurotrophic actions that remain less explored [29,30,31,37]. Recent studies indicate that SAC is associated with cell proliferation, suggesting that it could support both neuroprotection and post-stroke recovery [29,30,31,37]. For this reason, we examined its effects in young females over 15 days, during which endogenous neuroprotection and repair mechanisms are known to be activated [59,60].

SAC improved motor deficits and circling behavior, which could be associated with reduced infarct size, consistent with other preclinical reports showing that SAC attenuates post-ischemic motor deficits [61,62,63,64]. Clinically, motor deficits are an important determinant of post-stroke quality of life [65], especially in younger survivors who often experience difficulties returning to work [66].

Neurotrophin levels were evaluated as part of the neurprotective effect of SAC. BDNF is essential for the maintenance of several neuronal populations, including midbrain dopaminergic neurons, septal cholinergic neurons, and GABAergic neurons; it also modulates synaptic plasticity, enhances long-term potentiation (LTP), and prevents long-term depression (LTD). Spatial memory formation has been directly associated with increased BDNF levels [67]. In our study, SAC enhanced BDNF levels in the cortex and striatum, as assessed by IHC and WB, showing similar results and improved long-term recognition memory, without altering anxiety- or depression-like behaviors, suggesting that BDNF may contribute to the observed cognitive effects. VEGF intracerebroventricular promotes stroke recovery by enhancing neurogenesis and angiogenesis [68,69,70], and NGF also stimulate neurogenesis and angiogenesis after brain IR [70]. The increases in VEGF and NGF induced by SAC in the cortex and striatum also could be playing a role in post-ischemic repair mechanisms. The differences observed in VEGF levels using IHC and WB could be associated with the sensibility of each method, as IHC is less sensitive to modest global changes or may depend on region-of-interest selection and signal thresholding. It should be addressed in future work with additional markers and/or complementary quantification approaches.

SAC exposure has been reported to increase ERK phosphorylation and preserve BDNF in rat cortical slices, supporting neuroprotection under excitotoxic conditions [37]. Following cerebral infarction, the AKT and ERK pathways are rapidly engaged, and they remain activated for hours in peri-infarct regions, where they are associated with pro-survival and repair-related responses [71,72,73]. Consistent with this framework, AKT/ERK signalling contributes to neuronal survival and plasticity-related processes, whereas inhibition of these pathways is linked to increased apoptotic vulnerability [74,75]. BDNF/TrkB signalling is likewise implicated in post-stroke protection and recovery, and diverse interventions that enhance TrkB activation have been associated with improved outcomes, often involving downstream AKT/ERK engagement [76,77,78,79,80]. In this context, our findings show that SAC treatment increases phosphorylation of TrkB, AKT, and ERK in the cortex and striatum, suggesting a modulatory role for SAC in neurotrophin-related signalling during the post-ischemic period.

Additionally, the antioxidant properties of SAC may help maintain the redox balance required for neurogenesis and angiogenesis [81]. SAC has been reported to increase cell proliferation in healthy animals [32] and to promote neovascularization by activating the PI3K/AKT/eNOS pathway [31]. Activation of this pathway could, via CREB phosphorylation, contribute to the increased neurotrophin levels observed here, as reported previously [26].

Cell proliferation in response to stroke contributes to repair and regeneration. In male Wistar rats subjected to 1 h of ischemia, increased cell proliferation has been observed 1–2 weeks after reperfusion, as indicated by elevated Ki67 expression [82]. Neural progenitor cells have been detected two weeks post-reperfusion [83], and neurogenesis can persist for up to six weeks after IR [84]. Our data show that SAC treatment increased Ki67 expression in the cortex and striatum at this time window, suggesting that this compound could potentiate the trophic capacity induced by ischemia. However, future studies will be necessary to identify the specific cell types undergoing proliferation in response to SAC, as Ki67 is an inespecific proliferation marker.

Additional evidence supports a delayed maturation of proliferating cells. Fifteen days after global ischemia, proliferating cells have been detected in the subgranular zone without neuronal or astrocytic markers [85]; by 40 days, approximately 60% of these cells express neuronal markers and localize to the granular cell layer [85]. After focal ischemia, BrdU-positive cells have been described in the ipsilateral cortex up to 8 weeks after occlusion, with a peak at 2 weeks post-reperfusion [86]. Our results fit within this temporal profile and suggest that the increase in Ki67 induced by SAC could favor the generation of new cells that later integrate into damaged circuits.

Regarding hippocampal alterations, we observed a decrease in hippocampal BDNF and an increase in cortical and striatal BDNF after ischemia, suggesting that hippocampal BDNF migrates to the cortex and striatum to activate regenerative mechanisms. It has been shown that post-ischemic BDNF level is increased in peri-infarct regions and in the contralateral hemisphere during the first days after IR [87]. Such increases have been linked to the recruitment and directed migration of newly generated cells toward the lesion site, supporting neurogenesis and tissue remodeling. In this context, BDNF has been proposed to form molecular gradients that guide newborn neurons to damaged areas [88,89,90].

Similarly, we found that ischemia decreased VEGF mRNA in the hippocampus, suggesting a blunted pro-angiogenic response. VEGF is a key regulator of angiogenesis, and after cerebral ischemia, its expression has been reported to increase between 2 and 28 days post-injury, together with upregulation of its receptor, particularly in peri-lesional and contralateral regions [91]. By contrast, SAC has been shown to promote neovascularization after ischemic injury in muscle tissue [31], broadening interest in SAC as a potential modulator of post-ischemic vascular repair in the brain. Notably, despite the transcriptional changes, no significant differences in VEGF protein levels were detected among experimental groups. Additionally, discrepancies between mRNA and protein levels could reflect post-translational regulation and/or differences in protein stability and degradation. Studies at earlier time points could provide more information about increases in mRNA levels.

Clinically, anxiety symptoms are a frequent neuropsychiatric sequela after stroke and can negatively affect rehabilitation engagement and long-term functional outcomes [5]. Therefore, assessing anxiety-like behaviour in preclinical models provides translationally relevant information beyond motor and cognitive endpoints. With respect to anxiety-like behavior, previous studies have reported heterogeneous results depending on age, ischemia duration, and reperfusion time. In senescent male rats (24 months old), no increase in anxiety-like behavior was observed 10 days after MCAO [92]. In contrast, mice subjected to 90 min of ischemia exhibited increased anxiety-like behavior 72 h after reperfusion [93]. In young male and female mice, no significant changes in anxiety-like behavior were detected 15 days after reperfusion in the open field test [94]; however, mice subjected to 1 h of ischemia and 28 days of reperfusion showed increased anxiety-like behavior [95]. These findings suggest that anxiety symptoms may correlate with the extent of brain damage and the duration of reperfusion. In our study, no anxiety-like behavior was observed 8 days after reperfusion, but among animals subjected to MCAO, SAC produced anxiolytic-like effects. However, it has been reported that SAC reduced anxiety-like behavior in a chronic restraint stress at 21 days [96].

Post-stroke depression is another clinically relevant outcome with a complex and time-dependent trajectory. In the present study, depression-like behaviour did not differ significantly among groups at the assessed time point.

Future studies incorporating additional post-stroke time points (e.g., 30 days) and complementary behavioural tests will be valuable for determining the effects of IR and SAC.

Regarding memory, studies have shown that 28 days after MCAO, animals exhibit spatial learning deficits and memory impairments in the Morris water maze (MWM) and novel object recognition tests [95]. Two hours of ischemia followed by 15 days of reperfusion have been reported to decrease the discrimination index in the novel object recognition test, which evaluates long-term memory [97]. Young male rats subjected to 120 min of ischemia displayed acquisition memory deficits 8 days post-reperfusion, which were reversed by resveratrol, an antioxidant compound [98]. Similarly, animals exposed to 2 h of ischemia and 15 days of reperfusion showed deficits in the MWM [99]. In contrast, in our model, no statistically significant differences in MWM performance were detected, possibly due to reduced ischemic damage associated with the 1 h occlusion and the evaluation time point. It is also important to note that object recognition and MWM assess different types of memory. Object recognition is a non-spatial task that evaluates recognition memory, a component of declarative memory [100,101,102], whereas MWM primarily tests spatial memory and navigational ability, relying heavily on hippocampal integrity [103]. Our findings, which show that SAC improved long-term recognition memory but did not significantly affect spatial learning, are consistent with the region-specific effects of SAC described above, particularly the differential modulation of cortical/striatal versus hippocampal neurotrophin signaling.

The present study has several strengths. First, it addresses an underrepresented population in preclinical stroke research by focusing on young female rats and controlling the estrous cycle, thereby providing sex-specific mechanistic information that is often lacking in brain IR models. Also, the estrous cycle was monitored to ensure that all animals were at the same baseline point. Second, the selection of a time in the subacute phase (15 days after IR), a time window in which endogenous repair processes are active but still poorly characterized. While the aim of the acute neuroprotective interventions is to limit initial tissue damage, the 15-day post-ischemia assessment employed here captures processes related to functional recovery and brain plasticity, using histological, behavioral, and molecular endpoints. In particular, we evaluated infarct area, motor and cognitive outcomes, neurotrophin-related signalling (BDNF, NGF, VEGF, and TrkB/AKT/ERK), and cell proliferation providing a more integrated view of SAC effects on post-ischemic neuroplasticity. Third, the experimental design incorporated randomization, blinded outcome assessment, and quantitative analysis using two independent histological stains (H&E and Nissl), yielding convergent results that strengthen the internal validity and robustness of the findings. We further emphasize the administration regimen used in this study. In prior investigations that use SAC as neuroprotective agent, the compound was administered before IR injury, whereas in the present work, SAC was administered immediately before the initiation of reperfusion and after ischemic injury. Fourth, the use of well-characterised SAC dosing and administration protocols, based on previous ischemia studies, facilitates comparison with the existing literature and provides a rationale for future translational work exploring SAC as an adjuvant strategy in stroke recovery.

Moreover, we identify the limitations of our work. First, our study was limited to females; as we did not include a parallel male cohort, we cannot determine whether the observed SAC effects are sex-specific. In addition, future studies should include animals at different estrous stages to assess whether these effects extend across the whole cycle, and additional monitoring could be performed during the following days of reperfusion. Second, we cannot establish a direct causal relationship between SAC-induced changes in TrkB/AKT/ERK signaling, neurotrophin levels, cell proliferation, and behavioral outcomes because pharmacological inhibition of these pathways was not performed. Third, we evaluated only a single post-ischemic time point (15 days); therefore, the temporal dynamics of proliferative responses and behavioral alterations at earlier and more chronic phases must be determined. Fourth, sample sizes were modest and, in some behavioral assays, effectively lower (variable *n* across tests), which may have limited the detection of subtle effects. Fifth, we used a single SAC dose and administration schedule, so we could not define dose–response relationships or the therapeutic time window of SAC after ischemia. Sixth, we also quantified proliferation with Ki67 but did not determine the phenotype or long-term fate of the proliferating cells, which precludes firm conclusions about their contribution to neurogenesis versus gliogenesis. Seventh, all experiments were performed in otherwise healthy young female rats, which restricts the generalization of our findings to male and aged animals, as well as to stroke patients with common vascular and metabolic comorbidities.

A broad range of neuroprotective agents has shown efficacy in preclinical stroke models but failed to translate into clinical benefit, often due to limited therapeutic windows, heterogeneous patient populations, differences in age and comorbidities, and challenges in achieving adequate target engagement and dosing in humans. In this context, SAC has shown an important role in disease therapeutics models and in functional nutrition because it offers several practical advantages as a candidate to explore, including its high bioavailability, favorable safety profile as a garlic-derived compound, relatively low cost, and, as shown in this work, its beneficial post-ischemic effect on recovery. Moreover, the region- and domain-specific pattern observed here suggests that SAC may modulate selected repair-related pathways rather than producing nonspecific global effects. Nevertheless, overcoming historical translational barriers will require additional work in clinically relevant models (including aged animals, comorbidities, and both sexes), dose–response and pharmacokinetic–pharmacodynamic characterization, and ultimately controlled clinical trials to define effective dosing and timing.

## 5. Conclusions

In conclusion, SAC exhibits neuroprotective effects, including improved motor and cognitive performance and reduced infarct area, which could be associated with increased neurotrophin levels and proliferation and with activation of TrkB, AKT, and ERK signaling. Notably, these effects were region-specific, with greater central modulation in the cortex and striatum than in the hippocampus. These findings support SAC’s potential as a post-stroke therapeutic adjuvant; clinical studies are necessary to determine effective dosing and timing in humans. In addition, the present study was limited to female animals and evaluated a single dose at a single post-ischemic time point; future studies should assess sex as a biological variable, dose–response relationships, and temporal dynamics.

## Figures and Tables

**Table 1:** Number of animals used in each group.

Group	HA/IHC	WB	RT-PCR
SHAM	*n* = 3	*n* = 6	*n* = 4
SAC	*n* = 3	*n* = 6	*n* = 4
IR	*n* = 4	*n* = 6	*n* = 4
IR + SAC	*n* = 6	*n* = 6	*n* = 4

HA: histological analysis, IHC: immunohistochemistry, WB: Western blot, RT-PCR: reverse transcription polymerase chain reaction, SAC: S-allyl cysteine, IR: ischemia/reperfusion.

**Table 2:** Characteristics of the tests performed to evaluate motor function.

	Procedure	Normal Motor Function (Value = 0)	Altered Motor Function (Value = 1)
Mobility assessment	The animal is placed on a flat surface.	The animal actively explores its surroundings.	The animal remains immobile.
Contralateral grasp capacity	The animal is held by the tail and brought close to a rope suspended 30 cm above the ground.	The animal grips the rope with both forelimbs.	The animal can only grip with the ipsilateral forelimb.
Forelimb strength test	The animal is held by the tail and brought close to a rope suspended 30 cm above the ground.	The animal maintains its grip for more than 5 s.	The animal fails to grasp the rope with both forelimbs and releases it within 5 s.
Rotational behavior test	The animal is lifted by the base of the tail just enough to keep its forelimbs in contact with the surface.	The animal propels its body forward.	The animal exhibits rotational movements toward the contralateral side.
Forelimb extension reflex	The animal is lifted by the base of the tail, and it has no contact with the table.	Upon lifting, the animal extends both forelimbs.	The animal fails to extend the contralateral forelimb, either retracting it or exhibiting repeated contraction movements.

**Table 3:** The primers’ sequences used to evaluate gene expression (sequence 5’-3’).

Gene	Forward	Reverse
*BDNF* (NM_001270630.1)	AAAGGCATTGGAACTCCCAG	ATCCTTATGAATCGCCAGCCA
*NGF* (XM_039102402.1)	CGTACAGGCAGAACCGTACA	GAGGGCTGTGTCAAGGGAAT
*VEGF* (NM_001287114.1)	GTCACCGTCGACAGAACAGT	GACCCAAAGTGCTCCTCGAA
*ACTB* (NM_03114.3)	GATCAGCAAGCAGGAGTACGA	AACGCAGCTCAGTAACAGTCC
*GAPDH* (XM_063285517.1)	CCCCAACACTGAGCATCTCC	GTATTCGAGAGAAGGGAGGGC

*BDNF*: brain-derived neurotrophic factor; *NGF*: nerve growth factor; *VEGF*: vascular endothelial growth factor; *ACTB*: beta-actin; *GAPDH*: glyceraldehyde-3-phosphate dehydrogenase.

## Data Availability

The data presented in this study are available on request from the corresponding author. The data are not publicly available because they are stored on a personal server with limited storage capacity; therefore, they cannot be kept permanently available online. Behavioral recordings, photographs, and Western blot images require substantial disk space.

## Supplementary Materials

The following supporting information can be downloaded at https://www.mdpi.com/article/10.3390/nu18020362/s1, Figure S1. Effect of S-allylcysteine (SAC) on infarct area after ischemia/reperfusion (IR) in female rats; Figure S2. Effect of S-allylcysteine (SAC) on mRNA neurotrophin expression after ischemia/reperfusion (IR) in female rats; Figure S3. Effect of S-allylcysteine (SAC) on neurotrophins, pTrkB, pAKT, and pERK levels in hippocampus after ischemia/reperfusion (IR) in female rats; Figure S4. Effect of S-allylcysteine (SAC) on anxiety-like behavior after ischemia/reperfusion (IR) in female rats.

## Abbreviations

The following abbreviations are used in this manuscript:

ACTB	beta-actin
AKT	Protein kinase B
ARRIVE	Animal Research: Reporting of In Vivo Experiments
ARE	antioxidant response element
BDNF	brain-derived neurotrophic factor
ERK	extracellular signaling-regulated kinase
[^18^F]FDG	2-[^18^F]fluoro-2-deoxy-D-glucose
HO-1	hemoxygenase 1
H&E	Hematoxyline eosine
IACUC	Institutional Animal Care and Use Committee
GAPDH	glyceraldehyde-3-phosphate
HPRT	hypoxanthine-guanine phosphoribosyl transferase
IGF	insulin-like growth factor
IHC	immunohistochemistry
IR	Ischemia-reperfusion
MCAO	Middle cerebral artery occlusion
microPET	Micro positron emission tomography
NFkB	nuclear factor kappa-light-chain-enhancer of activated B cells
NGF	nerve growth factor
Nrf2	nuclear factor erythroid 2-related factor 2
2D-OSEM	2D Ordered Sets Expectation-Maximization algorithm
O.D	Optical density
PAF	paraformaldehyde
pAKT	phospho-AKT
pERK	phospho-ERK
PhiP	carcinogenicity caused by 2-amino-1-methyl-6-phenylimidazo[4,5-b]pyridine
pTrkB	phospho-TrkB
rTPA	recombinant tissue plasminogen activator
RT-PCR	reverse transcription polymerase chain reaction
ROS	Reactive oxygen species
SAC	S-allyl cysteine
SUV	standardized uptake value
TrkB	Tropomyosin receptor kinase
VEGF	Vascular endothelial growth factor
WB	Western blot
