# Supplementary material for: Garlic-Derived S-allylcysteine Improves Functional Recovery and Neurotrophin Signaling After Brain Ischemia in Female Rats
Source: Nutrients. 2026 Jan 22;18(2):362. doi: 10.3390/nu18020362 (PMC12845283; doi:10.3390/nu18020362)
Supplement: Supplementary file 1 [file nutrients-18-00362-s001.zip › nutrients-4090061-supplementary.pdf]

Article

Garlic-derived S-allylcysteine improves functional recovery and neurotrophin signaling after brain ischemia in female rats

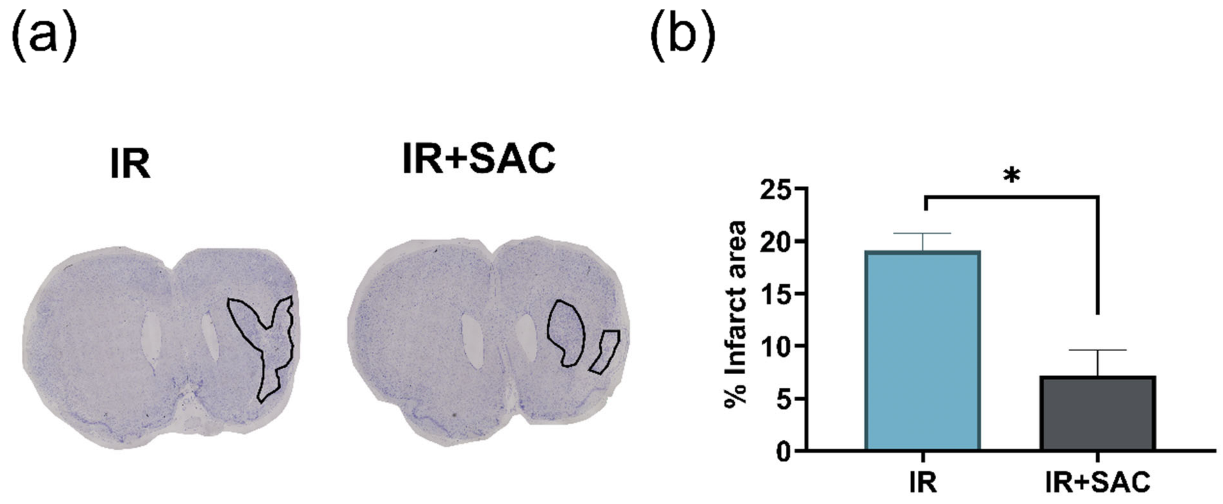

Figure S1. Effect of S-allylcysteine (SAC) on infarct area after ischemia/reperfusion (IR) in female rats. Animals underwent 1 h of ischemia followed by 15 days of reperfusion. **(a)** Representative coronal sections stained with cresyl violet (Nissl). **(b)** Quantification of the percentage of infarct area in the affected hemisphere by Nissl. Groups are: SHAM and SAC (n = 3 animals per group), IR and IR+SAC (n = 4). Data are represented as the mean ± SD. * $p < 0.05$.

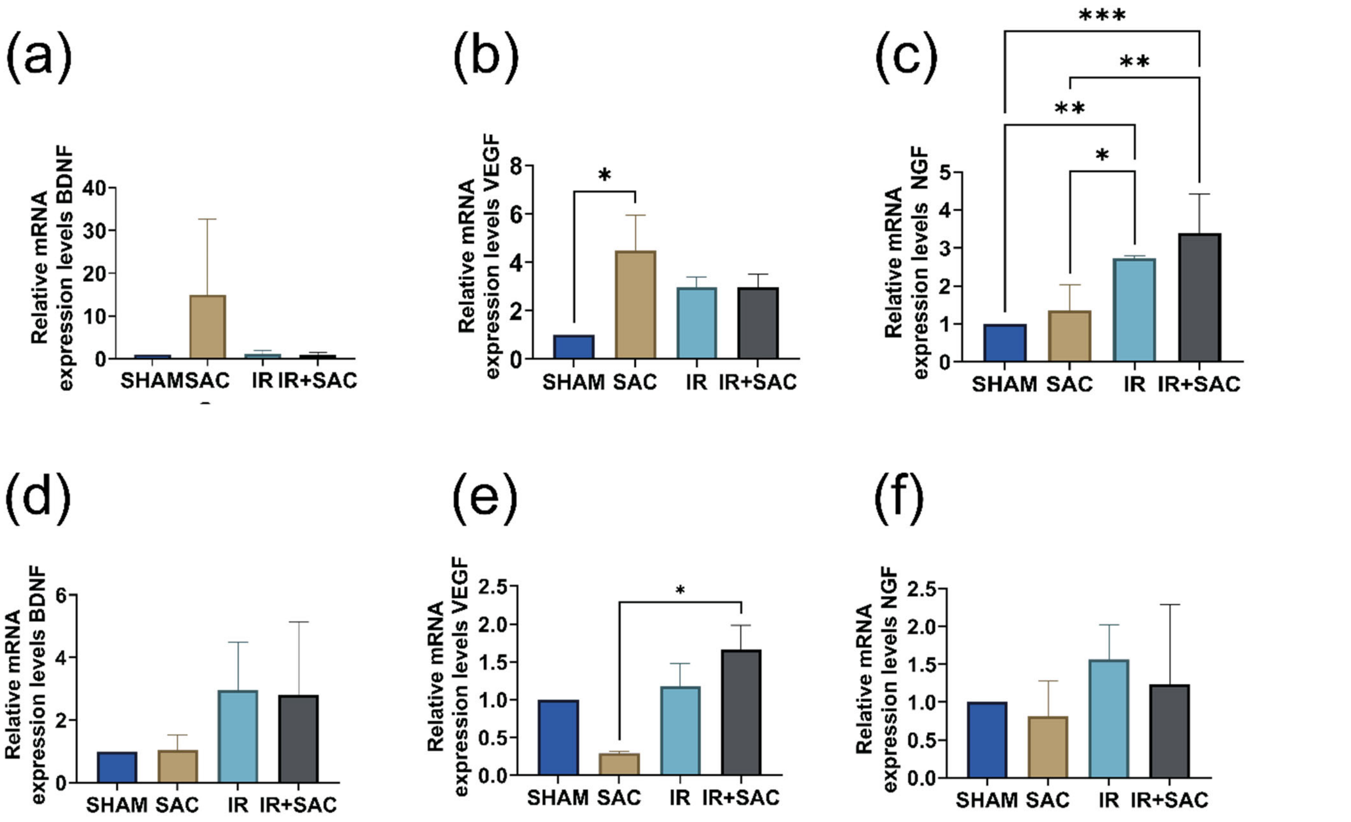

Figure S2. **Effect of S-allylcysteine (SAC) on mRNA neurotrophin expression after ischemia/reperfusion (IR) in female rats.** Animals underwent 1 h of ischemia followed by 15 days of reperfusion. Relative mRNA levels (RT-qPCR) for **(a)** BDNF, **(b)** VEGF, and **(c)** NGF in cortex and **(d)** BDNF, **(e)** VEGF, and **(f)** NGF in striatum are shown. SHAM, SAC, IR, and IR+SAC (n = 4 animals per group). Data are represented as mean ± SD. * $p < 0.05$, ** $p < 0.01$, and *** $p < 0.001$.

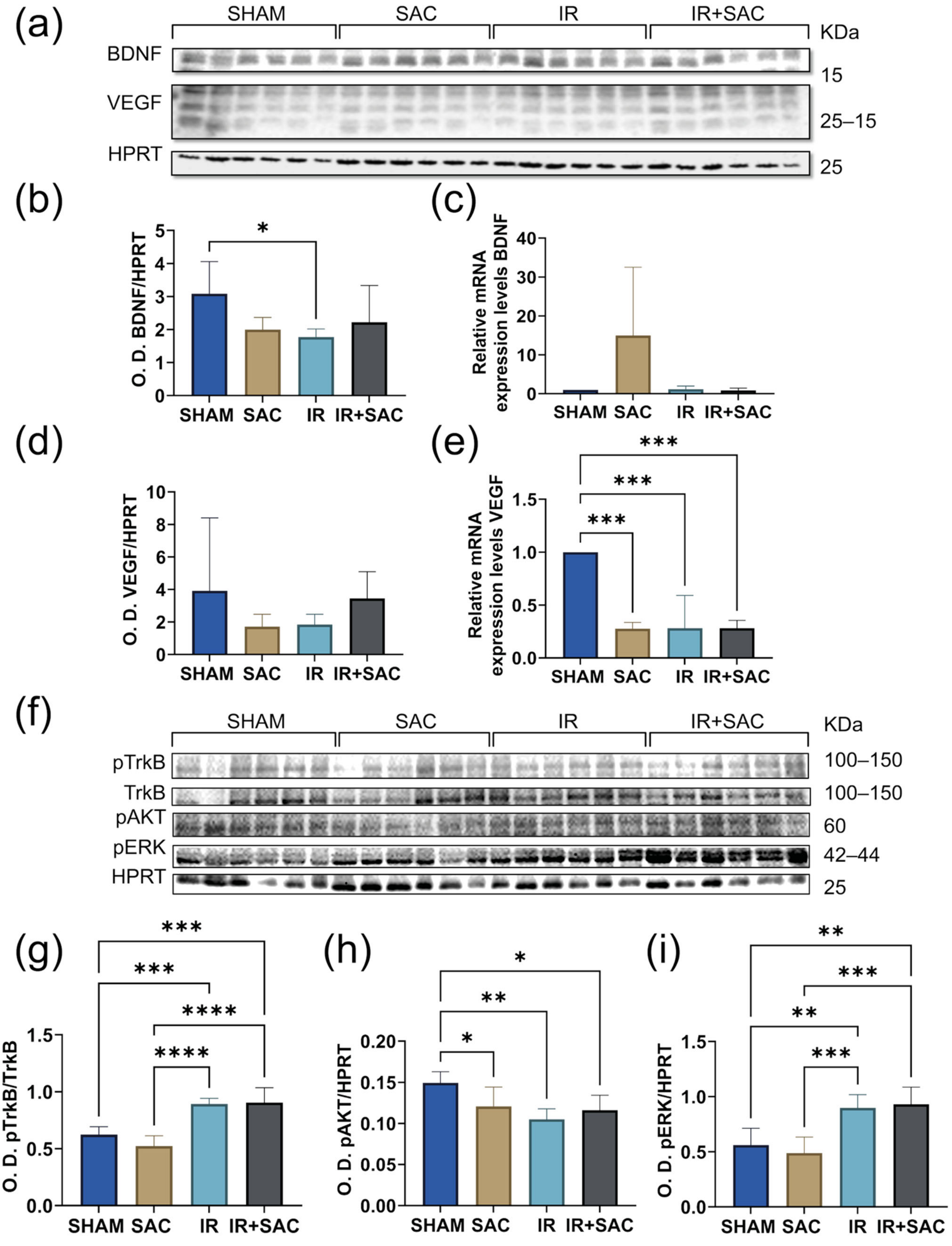

(a)
SHAM
SAC
IR
IR+SAC
KDa
BDNF
15
VEGF
25–15
HPRT
25
(b)
O. D. BDNF/HPRT
SHAM SAC IR IR+SAC
(c)
Relative mRNA expression levels BDNF
SHAM SAC IR IR+SAC
(d)
O. D. VEGF/HPRT
SHAM SAC IR IR+SAC
(e)
Relative mRNA expression levels VEGF
SHAM SAC IR IR+SAC
(f)
SHAM
SAC
IR
IR+SAC
KDa
pTrkB
100–150
TrkB
100–150
pAKT
60
pERK
42–44
HPRT
25
(g)
O. D. pTrkB/TrkB
SHAM SAC IR IR+SAC
(h)
O. D. pAKT/HPRT
SHAM SAC IR IR+SAC
(i)
O. D. pERK/HPRT
SHAM SAC IR IR+SAC

Figure S3. Effect of S-allylcysteine (SAC) on neurotrophins, pTrkB, pAKT, and pERK levels in hippocampus after ischemia/reperfusion (IR) in female rats. Animals underwent 1 h of ischemia followed by 15 days of reperfusion. **(a)** Representative Western blots (WB) of brain-derived neurotrophic factor (BDNF) and vascular endothelial growth factor (VEGF) in hippocampal lysates; hypoxanthine-guanine phosphoribosyltransferase (HPRT) was used as the loading control. Optical density (O.D.) for **(b)** BDNF and **(d)** VEGF. Relative mRNA levels by reverse transcription quantitative PCR (RT-qPCR) for **(c)** BDNF and **(e)** VEGF. **(f)** Representative WB of phospho-tropomyosin receptor kinase (pTrkB), tropomyosin receptor kinase B (TrkB), phospho-protein kinase B (pAKT), and phospho-extracellular signal-regulated kinases 1/2 (pERK) in hippocampal lysates. HPRT served as a loading control. O.D. for **(g)** pTrkB/TrkB, **(h)** pAKT/HPRT, and **(i)** pERK/HPRT. Data are represented as mean ± SD. SHAM, SAC, IR, and IR+SAC for WB (n = 6 animals per group) and mRNA (n = 4 animals per group). * $p < 0.05$, ** $p < 0.01$, *** $p < 0.001$, and **** $p < 0.0001$.

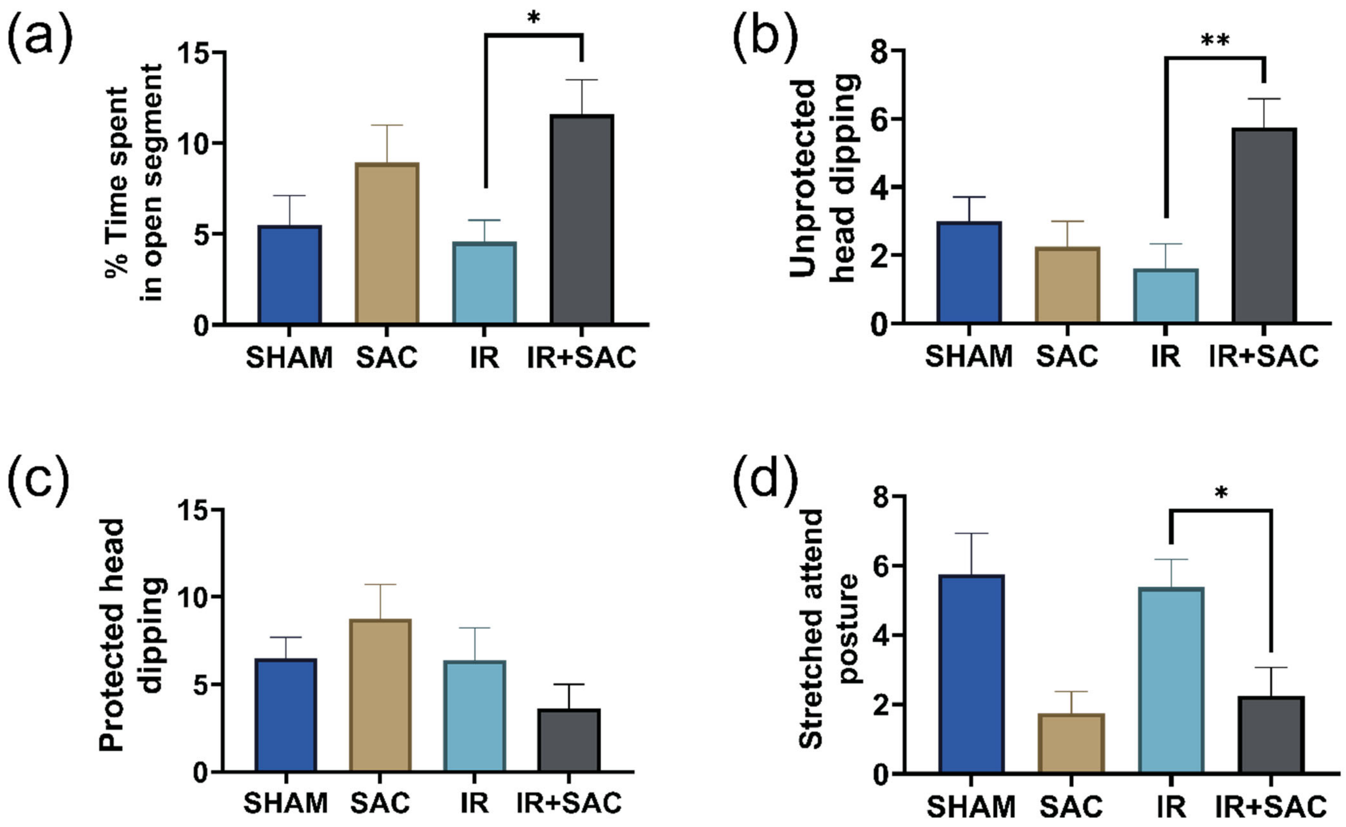

Figure S4. Effect of S-allylcysteine (SAC) on anxiety-like behavior after ischemia/reperfusion (IR) in female rats. Animals underwent 1 h ischemia and 15 days of reperfusion. The I-maze was performed on day 7 of reperfusion. **(a)** % time spent in open arm, **(b)** number of unprotected head dips, **(c)** number of protected head dips, and **(d)** number of stretch-attend postures. SHAM and SAC (n = 4 animals per group), IR and IR+SAC (n = 8 animals per group). Data are represented as mean ± SD. * $p < 0.05$ and ** $p < 0.01$.
